# Genome-wide association study identifies several loci for HEV seropositivity

**DOI:** 10.1016/j.isci.2023.107586

**Published:** 2023-08-10

**Authors:** Maria K. Smatti, Yasser A. Al-Sarraj, Omar Albagha, Hadi M. Yassine

**Affiliations:** 1 College of Health and Life Sciences, Hamad Bin Khalifa University, Doha, Qatar; 2 Qatar Genome Program, Qatar Foundation Research, Development and Innovation, Qatar Foundation, Doha, Qatar; 3 Centre for Genomic and Experimental Medicine, Institute of Genetics and Cancer, University of Edinburgh, Edinburgh, UK; 4 Biomedical Research Center, Qatar University, Doha, Qatar

**Keywords:** Association analysis, Immune response, Quantitative genetics, Virology

## Abstract

Hepatitis E viral (HEV) infection imposes a heavy global health burden. The variability in the prevalence of serological markers of HEV infection between different ethnic groups proposes a host genetic influence. Here, we report genetic polymorphisms associated with anti-HEV antibody positivity and level using binary- and quantitative-trait genome-wide association studies (GWAS) on a population from Qatar (n = 5829). We identified a region in 12p11.1 (lead SNP: rs559856097, allele: A, p *=* 2.3 × 10^−10^) significantly associated with anti-HEV antibodies level. This intergenic variant is located near SNORD112, a small nucleolar RNA (snoRNA). Additional gene-set and pathway enrichment analyses highlighted a strong correlation with anti-viral response-related pathways, including IFNs (alpha/beta) and interleukin-21 (IL-21) signaling. This is the first GWAS on the response to HEV infection. Further replication and functional experimentation are warranted to validate these findings.

## Highlights

• The impact of host genetics on HEV remains largely unknown
• GWAS identified a locus (12p11.1, rs559856097) associated with HEV antibody level
• GWAS genes were significantly enriched for anti-viral response pathways

## Graphical abstract

**Figure:**
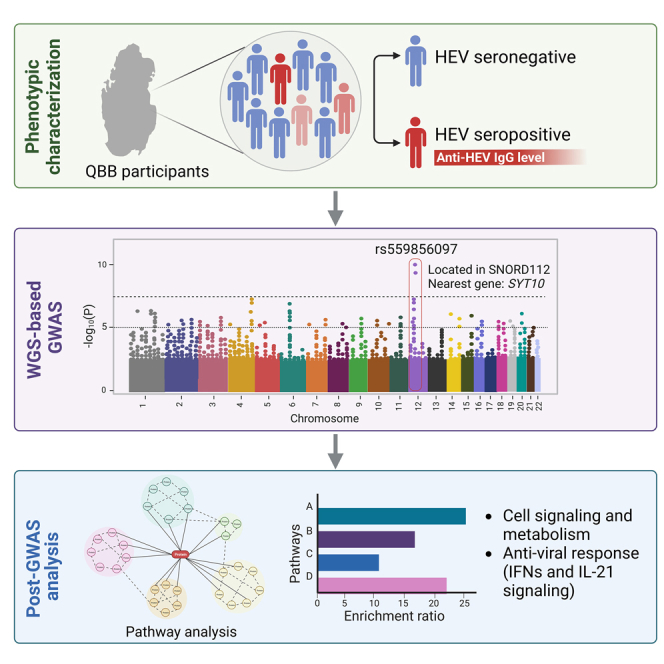

## Introduction

Hepatitis E virus (HEV) is a long-neglected RNA virus belonging to the *Hepeviridae* family. It is a major causative agent of acute viral hepatitis and jaundice cases worldwide.^1^ HEV is an emerging zoonotic virus with a broad host range including humans, pigs, dogs, rhesus monkeys, and many others.^2^ Hepatitis E is a serious public health concern especially in developing countries. The virus primarily causes acute infections in young adults, with a mortality rate reaching 2%. Infection with HEV is usually asymptomatic or self-limited in immunocompetent individuals, but can progress to chronicity and lead to fulminant hepatitis in immunocompromised patients and other risk groups such as pregnant women.^3^ High HEV-related morbidity and mortality rates are reported among pregnant women, infants <2 years old, and solid organ transplant recipients.^4^ Up to 20–25% of pregnant women can die if they get hepatitis E in the third trimester according to the WHO estimates.^5^

Globally, at least 20 million HEV infections occur every year, accounting for approximately 3.3 million symptomatic cases of acute illness. In a relatively recent study, it has been estimated that 110 million individuals have recent/current infection based on anti-HEV IgM antibody positivity, while 15 million carry an active infection according to the HEV RNA positivity.^6^ The prevalence of HEV varies between different geographical regions. Developed counties tend to have a lower HEV seroprevalence (5–20%) compared to developing countries, reaching almost 70% in HEV endemic regions.^7^^,^^8^^,^^9^ Similarly, variable rates of HEV were recorded in the Middle East and North Africa (MENA) region, ranging from 10% in Saudi Arabia and Tunisia to 90% in Sudan.^10^^,^^11^ In Qatar, a seroprevalence rate of approximately 21% was reported in blood donors, indicating a high HEV infection rate in the general population.^12^ The diversity of the people residing in Qatar and the high influx of foreign expatriates contribute to this high prevalence. In Qatari nationals, a prevalence of 11.5% was reported.^12^ Further, HEV IgM antibodies and viral RNA were detected in about 0.6%, supporting reported data from other countries about the risk of blood transfusion-associated HEV, particularly to pregnant women or immunocompromised patients.

Besides the environmental, behavioral, and socioeconomic factors, the variability in the prevalence of serological markers of HEV infection between different ethnic groups proposes a genetic basis for HEV susceptibility. A study in the US indicated that Hispanic ethnicity is associated with a higher seroprevalence of HEV compared to non-Hispanic blacks and non-Hispanic whites.^13^ Interestingly, it was also reported that non-Hispanic blacks carry ε3 and ε4 alleles in the *Apolipoprotein (APOE)* gene, which is associated with lower seroprevalence of HEV in this racial group, compared to non-Hispanic whites or Mexican Americans.^14^ In a replication study conducted on the Chinese Han population, Gu et al. reported that *APOE* variant was significantly associated with lower odds of anti-HEV seropositivity.^15^ Nonetheless, these findings require additional large-scale replication with particular attention to various confounders, especially since the role of APOE in HEV infection was not validated by *in vitro* experiments.^16^^,^^17^ A polymorphism in *Toll-like receptor 4 (TLR-4)* Thr399Ile (rs4986791) was also associated with HEV infection in an Indian cohort.^18^ Although these reports, to a limited extent, provided the first evidence that HEV infection could be modulated by host genetics, they were limited by the modest sample sizes and experimental design. A very small number of SNPs (around 1–500) were genotyped and correlated to HEV. Until now, no genome-wide studies have analyzed variants related to HEV infection at a whole genome level. The impact of host genetics in HEV infection remains largely understudied compared to other hepatic viruses, including Hepatitis C and B, which were extensively studied in previous GWASs. Most importantly, genomic studies are ancestry specific and need to be conducted on different populations to identify ancestry-relevant risk variants. In this study, we conducted a GWAS to identify genomic variants in the genomes of 6000 Qatari individuals that associate with antibody response to HEV infection.

## Results

### Seropositivity of anti-HEV IgG

Serological screening indicated a 12.2% (733/5998) positivity rate of anti-HEV IgG. HEV positivity was significantly higher among females (13%) compared to males (10.9%) (p = 0.018). In addition, a statistically significant positive correlation was found between anti-HEV IgG levels and age (p < 0.0001) (Table 1). Further classification of positive samples (n = 733) according to the ELISA reading indicated that most samples (n = 497) had an intermediate antibody level (groups 2 and 3), while 142 and 94 samples were considered strong and weak positives, respectively (Figure S1).

**Table 1:** HEV seroprevalence in the studied cohort

Category	No. (%)	Positive anti-HEV IgG	p value
Gender			
Male	2593 (43.2)	284 (10.9)	0.018
Female	3407 (56.8)	442 (13)	
Age groups			
15-30	1694 (28.2)	49 (2.9)	<0.0001
31-40	1624 (27.1)	62 (3.8)	
41-50	1378 (22.9)	144 (10.4)	
>50	1304 (21.7)	478 (36.7)	
Total	6000 (100)	733 (12.2)	

### SNP-level association analysis of HEV seropositivity

**Figure 1:**
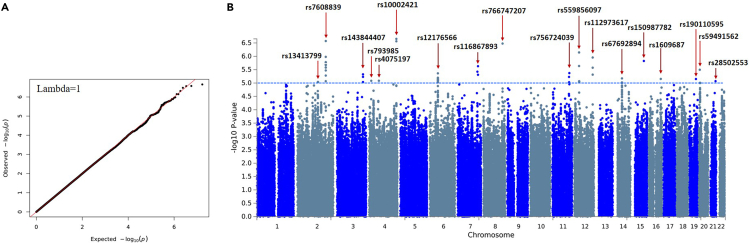
Quantile-Quantile (QQ) and Manhattan plots of the GWAS on HEV seropositivity binary trait (A) QQ plot of the association test statistics. The regression line (in red) represents the expected distribution of p values under the null hypothesis. Lambda value is the genomic inflation factor calculated as the ratio of the median of the observed distribution of the test statistic to the expected values. (B) Observed −log10 p values (y axis) are shown for all SNPs on each autosomal chromosome (x axis). The blue dotted line indicates suggestive significant of p value <1× $10^{-5}$. The top SNPs at each suggestive genomic loci are labeled with rs identifiers.

The binary trait association, which correlates anti-HEV IgG positivity with genomic variants, revealed 18 suggestive loci (p < 10^−5^). The strongest association was detected at 4q35.1 locus, where two SNPs (rs10002421 and rs4861528) showed suggestive evidence of association with HEV status (p = 2.2 × 10^−7^ and p = 8.2 × 10^−6^, respectively). The lead-independent SNP at this locus is rs10002421 (effect allele: G). Another association signal was also detected at chromosome 2, precisely at 2q32.2 (top lead SNP: rs7608839, p = 2.67 × 10^−7^), in addition to rs60915953, which was an independently associated variant with a p value of 1.05 × 10^−6^. The genomic locus 3q26.32 included two independent SNPs, rs143844407 (top lead SNP, p *=* 4.73 × 10^−6^) and rs190707108 (p *=* 9.1 × 10^−6^). Remarkably, the highest number of GWAS-tagged candidate SNPs was found in the genomic locus 6p12.1 (top lead SNP: rs12176566, p = 4.34 × 10^−6^, with 101 tagged SNPs). Moreover, one of the top SNPs of this association was derived by a single variant on chromosome 8 (rs766747207 at q24.21, p = 3.33 × 10^−7^). Similarly, we observed a relatively high association signal on chromosome 12 at p11.1 from two independent lead SNPs: rs559856097 and rs77221465 (p = 7.16 × 10^−7^ and p = 8.92 × 10^−6^, respectively). The q24.31 locus on chromosome 12 had also shown other suggestive association signals, with rs112973617 being the top variant (p = 1.1 × 10^−6^). Other variants at 15q25.1 (rs150987782), 7q34 (rs116867893), 7q33 (rs4728340), 20p12.3 (rs59491562), and 11q23.3 (rs756724039) showed suggestive evidence of association with HEV seropositivity (p value <5 × 10^−6^). Moreover, several loci with a marginal significant associations (*p* < 1 × 10^−5^) were detected at different chromosomes (Table 2; Figure 1).

**Table 2:** Top and independent SNPs at all genomic loci associated with HEV seropositivity binary trait

No	rsID	Chr	Pos	A1	A2 (effect)	AF_Allele2	AF (Cases)	AF (Controls)	Beta	Odds ratio	p-value	Nearest gene	Type of gene
1	rs7608839	2	190633678	T	C	0.82	0.77	0.83	−0.40	0.67	2.67E-07	NAB1	protein coding
2	rs60915953	2	190466578	C	G	0.85	0.80	0.85	−0.40	0.67	1.05E-06	MFSD6	protein coding
3	rs13413799	2	137888729	T	C	0.99	1.00	0.98	1.16	3.20	9.12E-06	AC020601.1	lincRNA
4	rs143844407	3	176225247	T	A	0.97	0.95	0.98	−0.82	0.44	4.73E-06	RP11-78E6.1	pseudogene
5	rs190707108	3	176213819	G	C	0.97	0.94	0.97	−0.74	0.48	9.18E-06	RP11-78E6.1	pseudogene
6	rs10002421	4	182853949	A	G	0.81	0.75	0.81	−0.39	0.68	2.22E-07	AC114798.1	miRNA
7	rs4075197	4	64697960	A	G	0.51	0.55	0.50	0.27	1.31	8.21E-06	RP11-158O16.1	pseudogene
8	rs793985	4	13926469	A	G	0.99	0.97	0.99	−1.06	0.35	8.24E-06	LINC01182	lincRNA
9	rs12176566	6	55741580	C	T	0.94	0.91	0.94	−0.56	0.57	4.34E-06	BMP5	protein coding
10	rs116867893	7	138958412	A	T	0.99	0.97	0.99	−1.29	0.28	2.35E-06	KIAA1549	protein coding
11	rs4728340	7	134850946	A	T	0.75	0.70	0.76	−0.32	0.72	3.76E-06	CALD1	protein coding
12	rs766747207	8	128517347	T	A	0.99	0.97	0.99	−1.36	0.26	3.33E-07	LINC00824	lincRNA
13	rs756724039	11	116068428	A	G	0.98	0.96	0.98	−0.93	0.39	4.24E-06	RPL5	pseudogene
14	rs238929	11	114101717	G	A	0.53	0.49	0.54	−0.27	0.76	5.91E-06	ZBTB16	protein coding
15	rs74355568	11	114453338	T	A	0.93	0.90	0.94	−0.52	0.59	9.33E-06	RP11-212D19.5	pseudogene
16	rs559856097	12	33204614	G	A	0.99	0.97	0.99	−1.37	0.25	7.16E-07	SNORD112	snoRNA
17	rs112973617	12	124927340	G	T	0.98	0.96	0.98	−0.97	0.38	1.11E-06	RPL22P19	pseudogene
18	rs77221465	12	33946298	T	C	0.99	0.98	0.99	−1.31	0.27	8.92E-06	RNU6-472P	snRNA
19	rs67692894	14	49337833	G	A	0.81	0.76	0.81	−0.34	0.71	6.71E-06	RP11-326E7.1	pseudogene
20	rs150987782	15	79305805	T	A	0.99	0.98	0.99	−1.50	0.22	1.49E-06	TMED3	protein coding
21	rs1609687	16	81402544	T	C	0.83	0.80	0.84	−0.37	0.69	7.01E-06	GAN	protein coding
22	rs190110595	19	44362376	C	T	0.98	0.99	0.98	0.92	2.51	7.08E-06	ZNF112:CTC-512J12.6	protein coding
23	rs59491562	20	5893391	A	AC	0.99	0.97	0.99	−1.25	0.29	3.18E-06	RNU1-55P	snRNA
24	rs73593909	20	7714039	T	A	0.82	0.78	0.83	−0.35	0.70	9.80E-06	AL031653.1	miRNA
25	rs28502553	21	41822335	C	G	0.77	0.72	0.78	−0.31	0.73	8.50E-06	PRDM15	protein coding

Chr: Chromosome number; Pos: Genomic position in GRCh38 hg38; A1: Allele1; A2, Allele2 that is the effect allele.AF: Allelic frequency.

### SNP-level association analysis of quantitative HEV seropositivity

**Figure 2:**
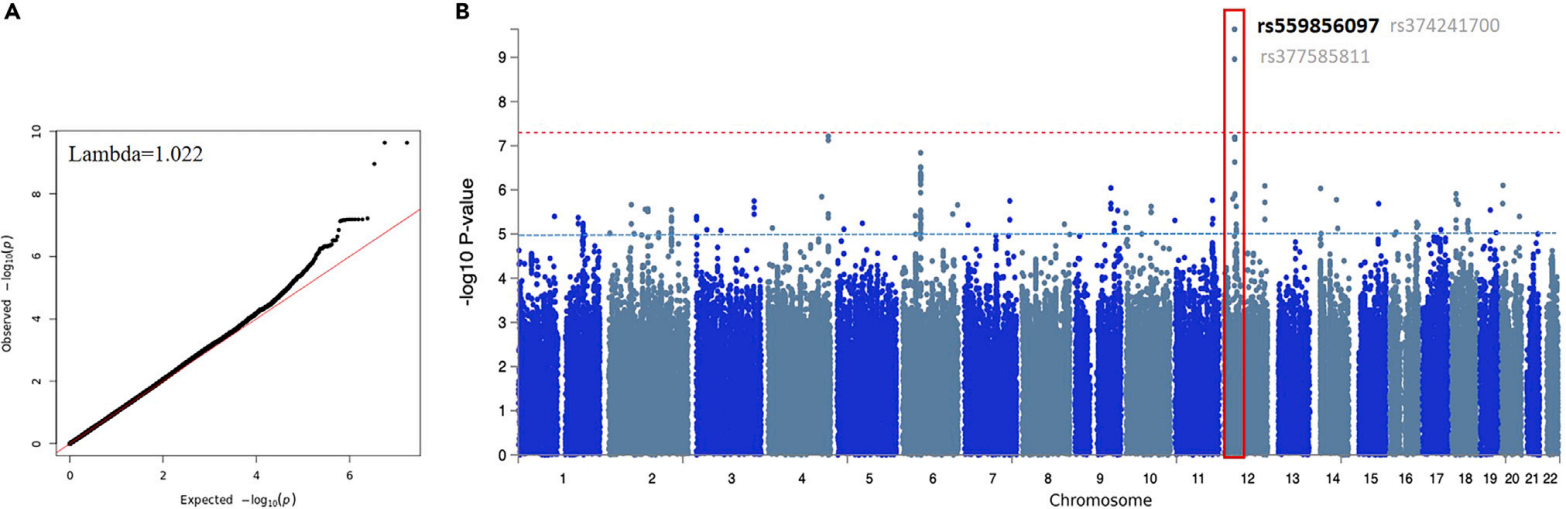
Quantile-Quantile (QQ) and Manhattan plots of the GWAS on anti-HEV antibodies level quantitative trait (A) QQ plot of the association test statistics. The regression line (in red) represents the expected distribution of p values under the null hypothesis. Lambda value is the genomic inflation factor calculated as the ratio of the median of the observed distribution of the test statistic to the expected values. (B) Observed −log10 p values (y axis) are shown for all SNPs on each autosomal chromosome (x axis). The red dotted line indicates GWAS significant of p value < 1× $10^{-8}$, while the black line indicates suggestive significant at p value < 1× $10^{-5}$. The GWAS significant genomic locus (12p11.1) is bordered with red, and the top SNPs are labeled with the rs identifiers. The top lead SNP is in black, while the two SNPs in the locus (dependent) are in light gray.

Genomic association with anti-HEV antibody level identified a single locus at chromosome 12 (p11.1) with genome-wide significance (p < 5 × 10^−8^). Three SNPs were detected at this locus, rs559856097 (the top lead independent SNP, p *=* 2.3 × 10^−10^), in addition to rs374241700 and rs377585811 (p = 2.3 × 10^−10^ and p = 1.09 × 10^−9^, respectively). Allele A of this lead variant (rs559856097) showed a negative correlation with anti-HEV antibody levels (Beta = −0.28). Importantly, SNPs at this locus were suggestively significant in the binary trait analysis, where rs559856097 was also detected as a lead SNP. Moreover, 16 unique candidate SNPs in LD with the independent significant SNP (rs559856097) were identified in this region. Of those, two SNPs were detected from this GWAS study (rs371520150 and rs372352546), while the remaining ones are known SNPs in the 1000G data (1KG/Phase3 reference panel) (Table S1; Figure 2).

Multiple other loci harbored variants close to the GWAS significance (*p* < 5 × 10^−7^), of which the majority have also been detected in the binary analysis. This includes the high signals observed at chromosome 4q35.1 locus (lead SNP: rs10002421, p *=* 6.08 × 10^−8^), and chromosome 6p12.1 (rs12176566, p = 1.44 × 10^−7^). The latter demonstrated the highest number of tagged SNPs (n = 101) compared to all other associated loci, in a similar pattern to that observed in the binary GWAS. Additionally, 61 suggestively significant SNPs (*p* < 1 × 10^−5^) were found at 56 genomic loci (Table S2).

### Gene mapping of the top GWAS SNPs

SNP annotation according to the physical position on the genome (positional mapping) at a 100 kb genomic window indicated that our GWAS SNPs are located within or near protein-coding genes, long intergenic non-coding RNAs (lincRNAs), microRNAs (miRNAs), pseudogenes, small nucleolar RNAs (snoRNAs), or small nuclear RNAs (snRNAs). The top hit of the binary trait association, rs10002421, is an intergenic variant located around 27.5 kb from AC114798.1 miRNA, and 64 kb from *DCTD* protein-coding gene. Additionally, the two SNPs at 2q32.2 were both linked to protein-coding genes. The variant rs7608839 is 13 kb close to *NAB1*, while rs60915953 is located within *MFSD6* genes. Other intronic SNPs positioned at protein-coding genes are rs116867893 and rs4728340 on chromosome 7, which were at *KIAA1549* and *CALD1*, respectively. Also, rs238929 is located within *ZBTB16*, rs190110595 in two overlapping genes, *ZNF112* and *CTC-512J12.6*, and rs28502553 is located in the *PRDM15* gene. Within a distance of 13 kb or less from a protein-coding gene, rs150987782, rs1609687, and rs12176566 intergenic variants were identified near *TMED3*, *GAN*, and *BMP5* genes, respectively (Table 2). The regional plots for all suggestive loci are presented in Figure S2.

**Figure 3:**
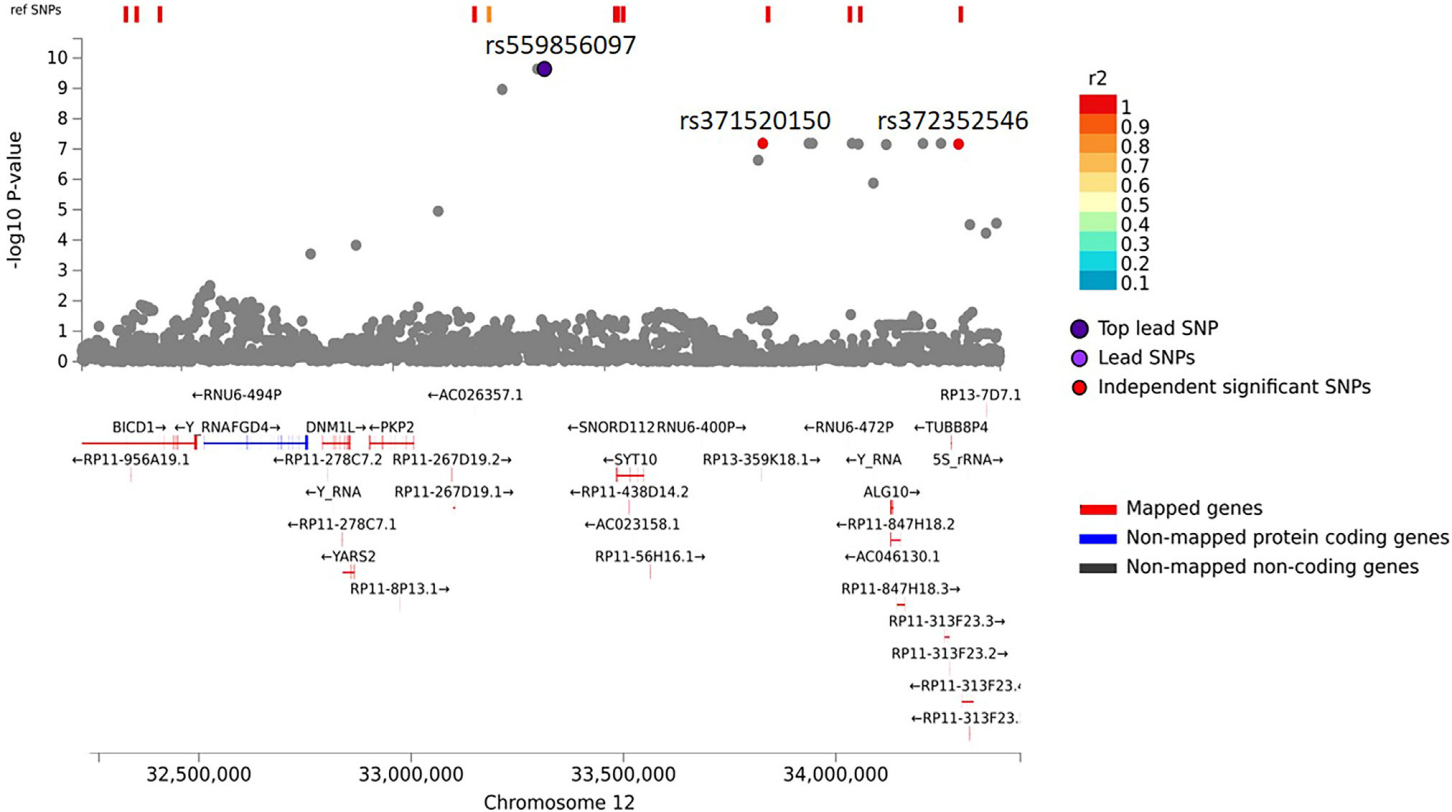
Regional association plot for the top signal at rs559856097 Genes were mapped to this locus by positional, eQTLs, and chromatin interaction mapping using FUMA platform (https://fuma.ctglab.nl/). The top lead and the independent significant SNPs are labeled with rs identifier.

On the other hand, rs559856097, the top SNP associated with quantitative HEV antibody response, is an intergenic variant located in SNORD112, a snoRNA on 12p11.1. The nearest protein-coding gene to this variant is *SYT10*, located after approximately 170 kb. The two independent SNPs at this locus, rs371520150 and rs372352546, were mapped to two RP11-313F23.4 (lincRNA) and RP13-359K18.1 (pseudogene), respectively (Figure 3).

In addition to positional mapping, eQTL and chromatin interaction mapping were performed. The complete list of mapped genes to all GWAS SNPs and SNPs in LD is presented in Tables S3 and S4. A list of 552 and 68 genes were mapped in the binary and quantitative GWASs, respectively. While no eQTLs were identified from the quantitative HEV GWAS, 26 out of 552 genes were mapped by eQTLs as shown in Table S5.

### Gene-set based analysis

**Figure 4:**
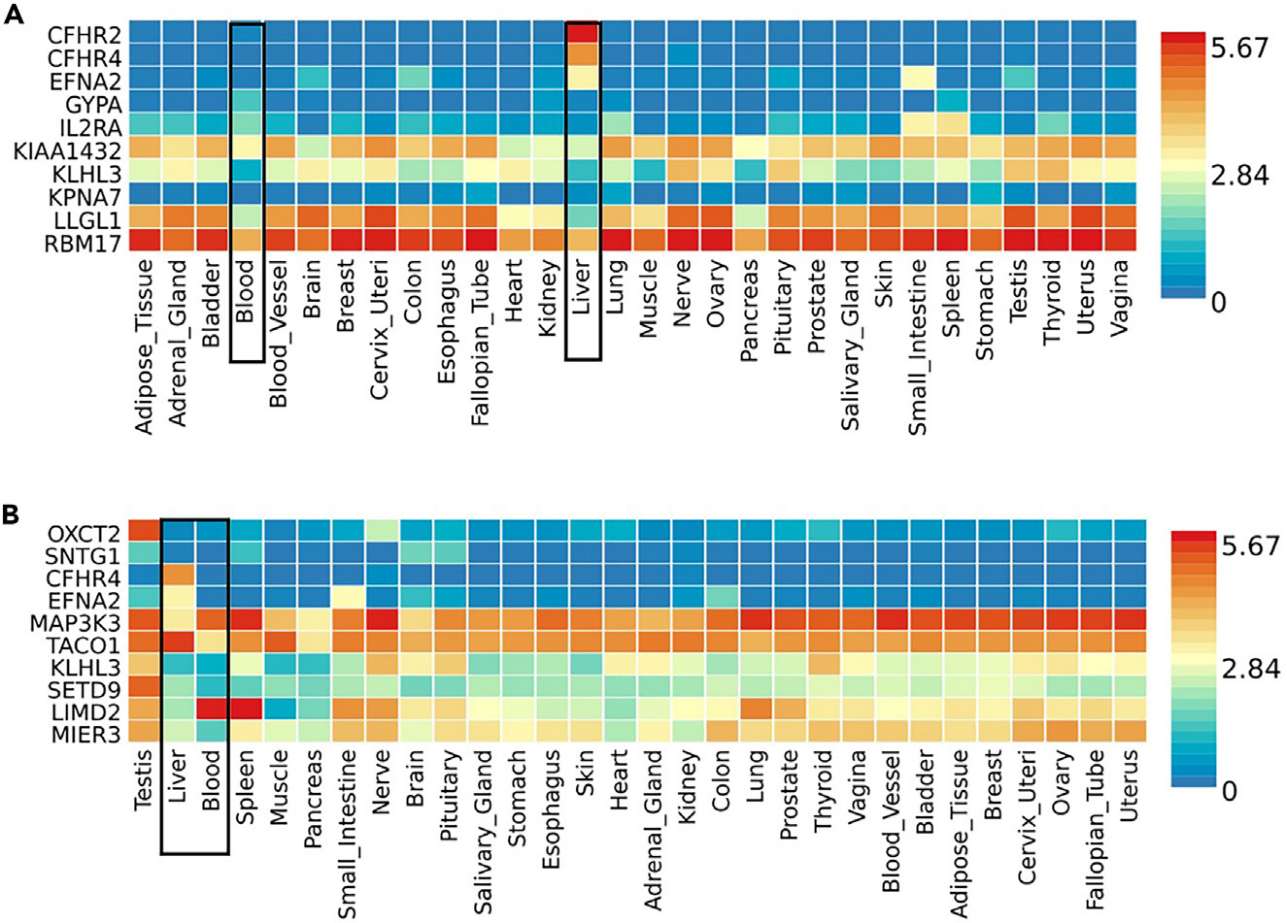
Heatmaps of the average gene expression per tissue (GTEx v8 30 general tissue types) for the top genes (highest p value) identified by MAGMA analysis (A) Top 10 MAGMA genes from the binary trait GWAS. (B) Top 10 MAGMA genes from the quantitative trait GWAS. The expression in blood and liver tissues are particularly bordered in black. FUMA platform (https://fuma.ctglab.nl/) was used for gene annotation and representation.

To further investigate the mechanisms underlying genetic signals, we performed gene-set-based analysis. We utilized the FUMA tool based on the Multi-marker Analysis of GenoMic Annotation (MAGMA). Initially, analysis of the binary trait GWAS genes revealed that among the top gene sets identified by MAGMA are inflammatory response, regulation SMAD2/3 signaling, antigen response, and PI3K/ErbB pathway gene sets (Table S6). Additionally, the top genes identified by MAGMA analysis included several genes involved in the immune system pathways, specifically, the innate immune response. This includes *IL2RA*, *KPNA7*, *CFHR4*, *KLHL3*, and *CFHR2* (Table S7). Moreover, these genes had a distinct expression in the liver compared to other tissues (Figure 4A). However, none of the identified pathways or genes had a false discovery rate (FDR) below 0.05. On the other hand, analysis of the quantitative GWAS mapped genes by MAGMA showed that the top gene sets are those related to PI3K/ErbB pathway, followed by nuclear import signal receptor activity pathway, and IRF3-mediated induction of type I IFN pathway (Table S8). While the first two pathways are generally related to cell signaling and general cell metabolism functions, the latter (IRF3 pathway) is one of the central pathways in regulating type 1-interferon during bacterial and viral infections. Additionally, among the top genes identified by MAGMA are genes closely related to interferon, class I MHC-mediated antigen processing and presentation, and complement pathways. This includes *MAP3K3*, *KLHL3*, *CFHR4,* and *CFHR2* genes. The list of the top ten MAGMA genes is presented in Table S9, while the tissue expression pattern is demonstrated in Figure 4B.

### Genes enrichment analysis

**Figure 5:**
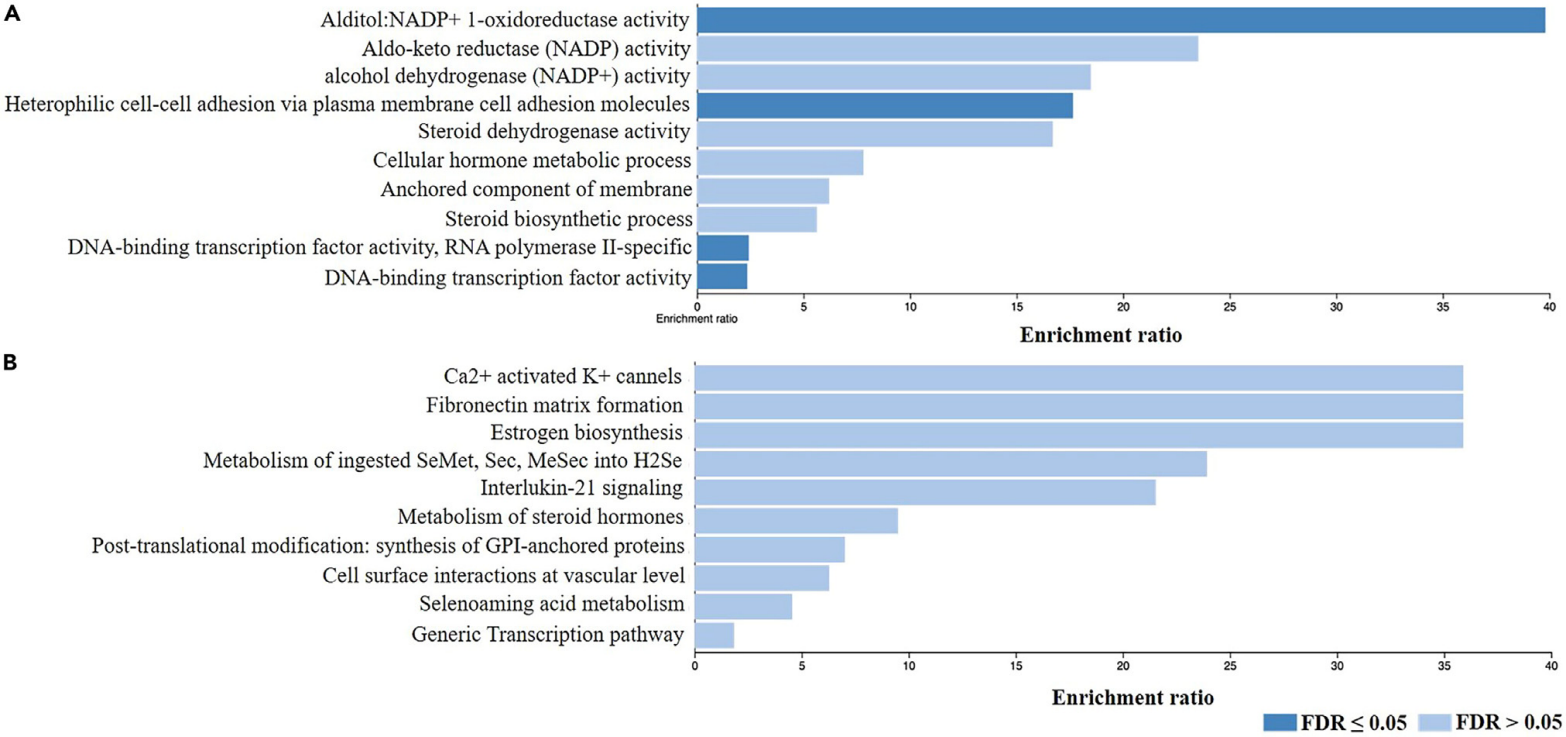
Gene set enrichment analysis of the binary trait HEV GWAS using WebGestalt (A) GO enrichment analysis on the candidate protein coding genes mapped by FUME on the binary GWAS gene set (n = 189 genes mapped from 18 genomic loci). (B) ORA pathway analysis using Reactome database on the same set of genes (n = 189). The top ten enriched pathways are presented in each bar graph. Categories with significant enrichment (FDR <0.05) are colored in dark blue.

To further analyze the biological roles of the mapped genes in our GWAS, we utilized WebGestalt, a functional enrichment analysis tool.^19^ We performed one of the main well-established methods for enrichment analysis, the over-representation analysis (ORA), in addition to gene ontology (GO) analysis. GO annotates genes based on cellular components (CC), biological processes (BP), and molecular functions (MF). GO enrichment analysis of the candidate protein-coding genes mapped from the binary GWAS (n = 189 genes mapped from 18 genomic loci), revealed several significantly enriched pathways, mostly related to metabolism and cell adhesion (*p* < 1 × 10^−6^, FDR <0.05) (Figure 5A). The ORA pathway analysis on the Reactome database showed that the most enriched pathways were those related to cell surface interactions at the vascular wall and Ca^2+^ activated K^+^ channels (*p* < 6 × 10^−5^, FDR = 0.054). The interleukin-21 signaling pathway was also one of the top enriched gene sets, with a suggestive significance (p value = 0.004, FDR = 0.8). The top enriched pathways of ORA Reactome analysis are presented in Figure 5B.

**Figure 6:**
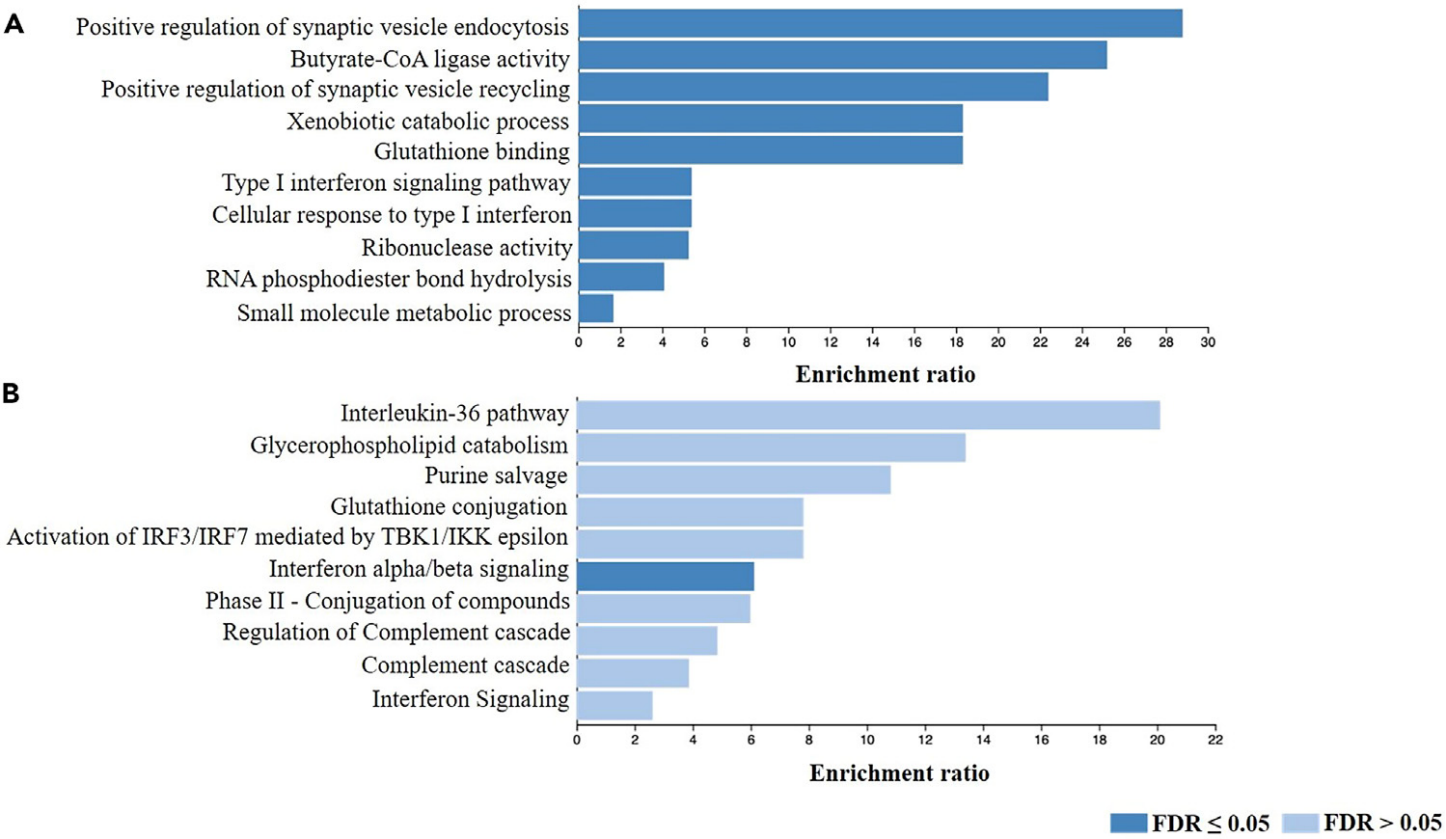
Gene set enrichment analysis of the quantitative trait HEV GWAS using WebGestalt (A) GO enrichment analysis on the candidate protein coding genes mapped by FUME on quantitate GWAS gene set (n = 441 candidate genes covering the significant and suggestive GWAS loci). (B) ORA pathway analysis using Reactome database on the same set of genes (n = 441). The top ten enriched pathways are presented in each bar graph. Categories with significant enrichment (FDR <0.05) are colored in dark blue.

In the quantitative GWAS genes enrichment analysis, 441 candidate genes covering the significant and suggestive GWAS loci were included. Of these, 379 genes were unambiguously mapped to the database. GO pathway analysis revealed multiple significantly enriched pathways (Figure 6A). While various gene sets were related to metabolic pathways, type I interferon signaling and cellular response signaling pathways were again significantly enriched (Both p value and FDR <0.05). Similarly, Reactome pathway analysis showed that the Interferon alpha/beta signaling pathway is enriched substantially (p = 1.4 × 10^−5^, FDR = 0.025), compared to interleukin-36, compliment, and metabolic pathways, which were among the top pathways but with a suggestive enrichment (p < 0.05, FDR >0.05) (Figure 6B).

**Figure 7:**
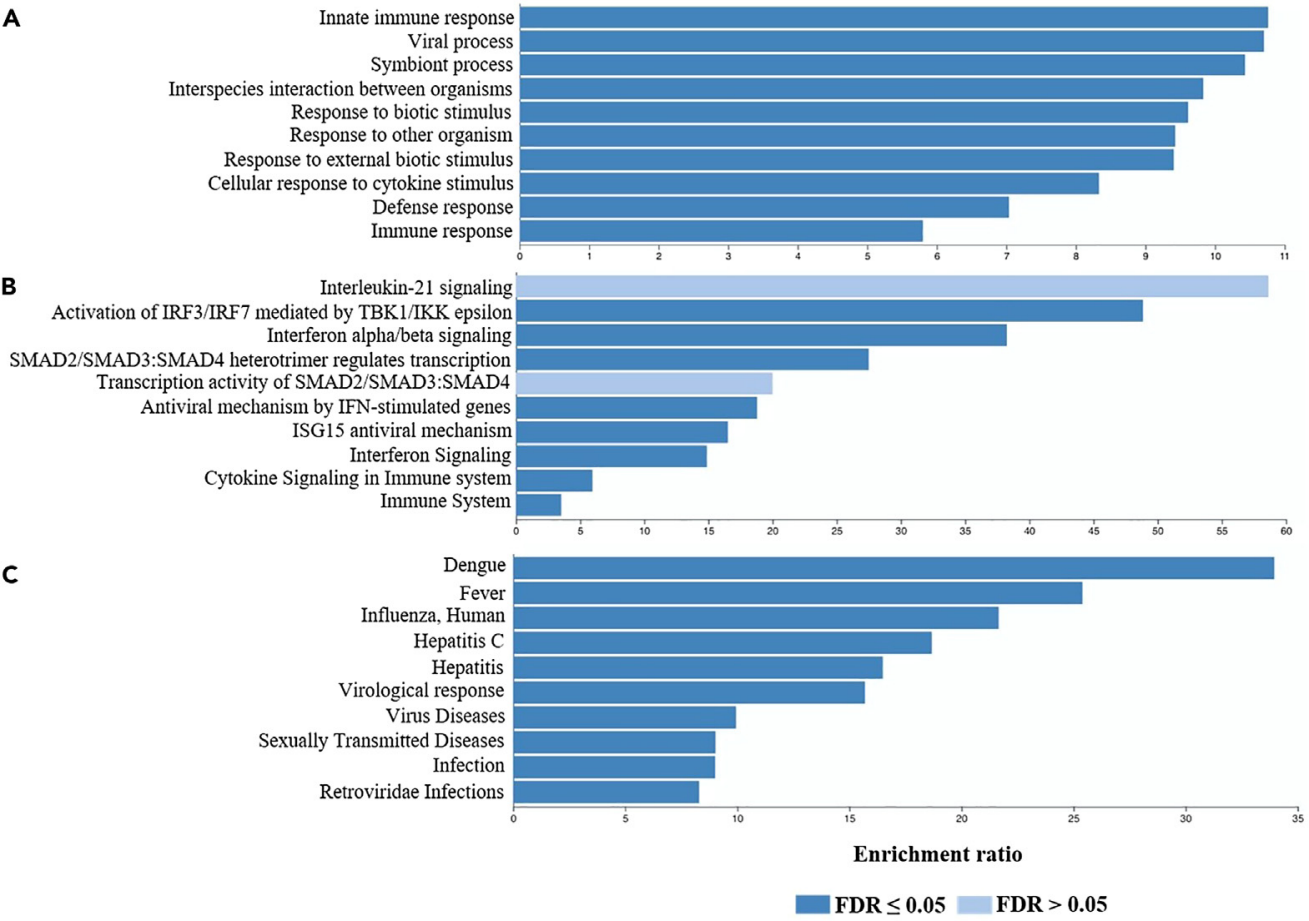
Gene set enrichment analysis of immune-related genes from the binary and quantitative trait HEV GWASs using WebGestalt (A–C) A list of 43 immune genes filtered from our candidate genes list was used for: A.GO enrichment analysis. B.ORA pathway analysis. C. ORA in GLAD4U disease database. The top ten enriched pathways are presented in each bar graph. Categories with significant enrichment (FDR <0.05) are colored in dark blue.

Considering that our GWAS is based on the phenotypic characterization (antibody response) of cases based on their immune status with regard to HEV, we further analyzed the results focusing exclusively on immune genes and pathways. Initially, candidate protein-coding gene sets identified from both binary and quantitative GWASs were combined. This resulted in a list of 537 unique genes included in the InnateDB database search. Of these, 43 genes were directly related to the immune response and were therefore subjected to further gene enrichment analysis. Starting with the GO gene sets, we found a highly significant enrichment in genes related to the innate immune response particularly (p = 1.×10^−16^, FDR = 1.36 × 10^−12^). Other immune-related pathways were also markedly enriched, including those pertaining to viral processes (Figure 7A). Reactome pathway analysis indicated that our candidate genes are enriched for several interferon and cytokine signaling, as well as anti-viral mechanisms pathways (*p* < 1 × 10^−4^, FDR <0.01) (Figure 7B). Besides, we utilized the GLAD4U database to explore the pattern of gene enrichment in disease-related pathways. The most significant enrichment was found in pathways predominantly related to viral infections (FDR = 2.9 × 10^−9^). Moreover, significant enrichment of pathways involving different infections was observed, including dengue, influenza, and sexually transmitted diseases. Notably, hepatitis-associated pathways were also enriched with high statistical significance (FDR <0.00005) (Figure 7C).

## Discussion

The role of host genetic factors in the susceptibility or response to infections is increasingly recognized. Nevertheless, genome-wide assessment of human genetic determinants of antibody responses to many viral infections is still scarce, including the response to HEV. In this present study, we mapped genetic loci associated with HEV infection in a 6,000 well-characterized cohort. This was accompanied by gene network analysis and gene overrepresentation to explore possible underlying molecular mechanisms.

We initially determined the HEV serostatus at a 12.2% positivity rate among the adult Qatari population. This prevalence is in line with previous reports, where an 11.5% seropositivity rate was reported in Qatari blood donors,^12^ and neighboring counties such as Saudi Arabia (11% in apparently healthy population). However, higher seroprevalence of anti-HEV antibodies was reported in other Middle East and North Africa (MENA) region, such as Egypt (47% in pregnant women) and Sudan 90%.^10^^,^^11^ We based our phenotypic classification on ELISA assay because of its sensitivity in determining the immune status. Importantly, the commercial kit (MP Diagnostics HEV ELISA) that was utilized in the study had a recorded sensitivity of 99.2%. Following the serostatus determination, we used two GWAS approaches, a binary and a quantitative phenotype, to pinpoint genomic regions associated with HEV seropositivity or antibodies level, respectively.

Prior exposure to HEV evidenced by a positive anti-HEV IgG test result was suggestively associated with 18 genomic loci. Most of the signals at these loci map to non-coding regions (15 out of 25 variants), which is expected for whole-genome association analyses.^20^ Additionally, ten variants were intronic or intergenic and mapped to a protein-coding gene. The nearest protein-coding genes to intergenic variants were *NAB1*, *BMP5*, *TMED3*, and *GAN*. On the other hand, intronic variants within protein-coding genes were found in *MFSD6*, *KIAA1549*, *CALD1*, *ZBTB16*, *ZNF112*, and *PRDM15* genes. Notably, most of these genes have a functionally validated or predicted role in regulating the immune response. For instance, *NAB1* is involved in the regulation of IFNGR1, the receptor for type II interferon (IFNγ), and thus, associated with susceptibility to various infections. Interestingly, previous studies demonstrated that polymorphisms in *ifngr1* promoter correlated with hepatitis B infection susceptibility.^21^ Hence, it is possible that variants of *NAB1*, a regulator or IFNGR1, could also play a role in susceptibility and/or immune response to HEV. *MFSD6*, *GAN*, and *ZBTB16* genes were also linked to class I MHC (MHC-1) antigen processing and presentation. In fact, the role of CD8^+^ T cells in combating viral infections is well studied. These immune cells function by recognizing viral-derived peptides presented by MHC-1 on the surface of infected cells.^22^ Manipulation of the MHC-1 presentation pathway has been found to limit the MHC-1 expression, reduce the ability of CD8^+^ T to recognize viral peptides, and thus, impair viral clearance.^23^ While the exact role of *MFSD6*, *GAN*, and *ZBTB16* genes in MHC-1 pathway is still not clear, mutations in genes involved in any process of MHC-1 expression or antigen processing and presentation pathways could alter the subsequent immune responses. Given the important role of MHC-1 molecules in detecting virally infected cytotoxic T lymphocytes (CTLs), multiple viruses evolved proteins that inhibit the MHC-1 pathway through diverse mechanisms for host immune impairment and viral evasion.^24^

Two ZNF protein-coding genes were also prioritized in our GWAS, *PRDM15*, and *ZNF112*. The ZNF protein family is known for its essential function in regulating immune responses at transcriptional and post-transcriptional levels.^25^ *PRDM15* gene, tagged by rs28502553 intronic SNP, is part of the immune response cluster as classified by the Human Protein Atlas. It is expressed in immune cells, overexpressed in B-cell lymphomas, and plays an essential role in B-cell lymphomagenesis.^26^ *ZNF112* gene, on the other hand, has been linked to immunoglobulin resistance in Kawasaki Disease (KD) possibly due to its regulatory contribution to inflammation.^27^ Importantly, KD could be triggered by various infectious agents in genetically susceptible individuals.^28^ Still, the role of *PRDM15* and *ZNF112* in the immune response to infections has never been studied. Therefore, further studies are needed before we understand the mechanistic role of polymorphisms in *PRDM15* and *ZNF112* on HEV antibody response.

*BMP5* and *TMED3*, the nearest protein-coding genes to rs12176566 and rs150987782, also have immune-related functions. While BMP5 acts primarily as a growth factor and is linked to cytokine activity, TMED3 belongs to the TMED family, an important player in innate immune signaling.^29^ Besides the involvement in immunity, other genes with diverse functions were also tagged by several suggestive SNPs. This includes *KIAA1549* and *CALD1*, which are associated with brain tumors and regulation of muscle contraction, respectively.

We noticed multiple signals from SNPs associated with HEV seropositivity at non-coding RNAs (ncRNAs), specifically the long intergenic ncRNAs. Generally, this class of non-coding RNAs has been recently described as critical regulators in anti-viral responses and virus-host interactions.^30^ Moreover, in a recent study on HBV integration sites, it was reported that the HBV genome was highly integrated into several non-coding RNAs, including LINC00824.^31^ This particular lincRNA was tagged by rs766747207, one of the top SNPs in our binary HEV GWAS. Although HBV is a DNA virus with the potential basis for host genome integration that resembles retroviral integration, non-retroviral RNA viruses can incidentally integrate.^32^ Nevertheless, the definitive role of LINC00824 and other ncRNAs identified in our study in HEV infection necessitates additional research.

In addition to long non-coding RNAs, we identified four SNPs in short non-coding RNAs regions, of which two were located at miRNAs (AC114798.1 and AL031653.1). Host-encoded miRNAs are known for their ability to modulate the host-virus interplay, given their pivotal role in various cellular processes. These miRNAs could either promote or repress viral replication by suppressing or upregulating the IFN-α/β signaling pathway.^33^ In fact, the link between cellular miRNAs and viruses emerged after the observation that HCV infection affects the expression of miRNA-122, which enhances HCV replication and the stability of its RNA.^34^^,^^35^ In HEV infection, several miRNAs (such as: miR-450b, miR-125b-5p, miR-192-5p, and miR-99a) have been identified and associated with HEV progression to chronicity or acute liver failure.^36^^,^^37^ However, until now, there have been no studies on the role of AC114798.1 and AL031653.1 miRNAs in HEV or any other viral infection.

Next, we investigated whether host genetic variants contribute to the level of antibodies in HEV infection. To count for the possible differences related to the time of exposure due to demographic differences, we adjusted our model for age and gender before conducting the quantitative GWAS. We identified a region in 12p11.1 (Lead SNP: rs559856097) significantly associated with anti-HEV levels. Carrying A allele negatively correlated with the level of anti-HEV antibodies. This strong GWAS signal came from a variant near SNORD112, a snoRNA. SNORDs are a highly expressed class of non-coding RNAs that play a prominent role in rRNA modification and act as a scaffold for protein complexes.^38^ Solid evidence has accumulated regarding the clinical role of SNORDs in disease outcomes, including viral infections. These nucleolar RNAs are found to interact with RNA viruses. In particular, a specific C/D box snoRNAs is needed for optimal viral replication, as observed with dengue fever virus, influenza A virus, human rhinovirus 16, herpes simplex virus 2, and others.^39^ In a similar way, C/D box 126 (SNORD126) has been recently reported to enhance HCV replication by activating the PI3K-AKT signaling pathway.^40^ However, SNORD112, is one of the orphan snoRNAs that exist with unknown complementarity to any cellular RNA and, thus, unknown function.^41^ Overexpression of SNORD112, in addition to SNORD113 and SNORD114, was documented in acute promyelocytic leukemia (APL).^41^ However, the precise role of SNORD112 and its involvement in viral infections remains open for investigation. The nearest protein-coding gene to the lead SNP (rs559856097) at 12p11.1 was *SYT10* gene. This gene acts as a calcium sensor needed explicitly for the Ca(2+)- dependent exocytosis of secretory vesicles containing insulin-like growth factor 1 (IGF1) in neurons of the olfactory bulb.^42^

Despite the diverse functions of the protein-coding genes mapped by the binary and quantitative GWASs, most of these genes carry polymorphisms associated with liver diseases and viral infections, as demonstrated in the GWASs PhenoScanner database. For instance, variants in the *BMP5* gene have been linked to acute respiratory infections, while *TMED3* gene variants were strongly linked to HBV. On the other hand, *MFSD6*, *CALD1*, *GAN*, and *PRDM15* genes were associated with UTIs. Theoretically, genetic variants in common innate and adaptive immune-related genes could interfere with the host response to different viruses regardless of their site of infection. On the other hand, liver cirrhosis, fatty liver, and hepatic failure phenotypes were associated with variants in *NAB1*, *CALD1*, *ZBTB16*, *GAN*, *TEMD3*, and/or *PRDM15* genes. Of note, the progression of HEV infection to chronicity, leading to liver damage and cirrhosis has been previously reported.^43^

In order to employ a more comprehensive analysis of genetic markers that simultaneously contribute to a complex phenotype, we applied MAGMA gene and gene-set analyses. Several gene sets prioritized by MAGMA were closely related to the immune response processes. This included genes of IRF3-mediated induction of type I IFN and SMAD2/3 signaling, which are essential pathways in regulating type 1 interferons during bacterial and viral infections.^44^^,^^45^ In fact, treatment by different types of interferons (type I, II, and III IFNs) demonstrated an anti-viral response to HEV infection.^46^ Moreover, HEV and other hepatitis viruses express molecules that target signal transductions across IFN pathways as a defensive mechanism to block the production of IFNs and impair the host anti-viral response.^47^^,^^48^^,^^49^ Interestingly, specific polymorphisms in *IFNλ4* gene were found to correlate with anti-HEV IgG positivity among hemodialysis patients.^50^ It is, therefore, not surprising that variants in genes involved in IFN signaling pathways could modulate the susceptibility, seropositivity, or response to HEV infection.

Further, we performed an overrepresentation enrichment analysis using several databases to identify pathways whose perturbation may contribute to the HEV infection. Remarkably, in addition to involvement in metabolism, our candidate genes were significantly enriched for interferons (alpha/beta) signaling and cellular response to IFN-1 pathways. This again highlights the possible key role of IFNs in HEV response. Furthermore, IL-21 signaling pathway was suggestively enriched in the quantitative GWAS candidate gene set. IL-21 is a major cytokine produced by T follicular helper (Tfh) cells. Along with other cytokines, IL-21 modulates antibody production by T-cell-dependent activated B-cells. It also enhances somatic hypermutation and production of high-affinity antibodies from plasma cells.^51^ Most importantly, the observed enrichment in IFNs and antibodies production pathways strengthens the validity of our GWAS findings since it was mainly designed to detect variants associated with antibodies positivity or level.

As a complementary approach to explore enriched pathways exclusively related to immunity, we further performed enrichment analysis on genes directly involved in the immune response. Across a list of 43 immune genes filtered from our candidate genes list, we identified highly enriched gene sets contributing primarily to anti-viral responses. This included anti-viral responses to various hepatic (e.g., HCV and HBV) and non-hepatic viruses (e.g., measles, influenza). Notably, this points out that polymorphisms in genes related to common anti-viral signaling pathways could modulate the response to distinct viruses from different groups. This also highlights the need for more genomic studies and, most importantly, meta-analyses combining GWAS findings on different infectious diseases. Determining common variants that disturb overlapping anti-viral response pathways could provide potential preventive, prognostic, and therapeutic targets for genetically predisposed individuals.

The strength of this study comes from the uniqueness of the Qatari population, which provided an opportunity to detect variants that contribute to HEV infection in genomes representing the Qatari and Arab regions, an extremely underrepresented population in genomic studies. A recent report on the genetic composition of the Qatari population included the same cohort as in the present study and identified six major ancestries: General Arabs (38%), Peninsular Arabs (17%), Arabs of Western Eurasia and Persia (22%), South Asian Arabs (1%), African Arabs (3%), and Admixed Arabs (19%).^52^ The richness in the genomic diversity of the population makes it a perfect representative of the whole Middle Eastern region. In fact, the unique population structure could partially explain why our results did not replicate previous reports on the role of *TLR-4* and *APOE* polymorphisms in HEV infection in non-Hispanic whites, non-Hispanic blacks, Mexican Americans, Indians, and Chinese populations.^14^^,^^15^^,^^18^ Zhang et al. study found that non-Hispanic blacks were the only population that showed an association between anti-HEV seropositivity and APOE variants, compared to other studied populations.^14^ Interestingly, APOE ε3 and ε4 variants were found to be significantly associated with protection against HEV infection in non-Hispanic blacks, who had the lowest seroprevalence of anti-HEV (15.3%) compared to other groups (>20%).^14^ This emphasizes the importance of ancestry-specific genomic studies to understand infections' susceptibility and pathogenesis at a population level. Additionally, this study was based on whole genome sequencing data rather than SNP array or targeted genotyping, and thus, was able to detect variants beyond the protein-coding regions. This allowed for a comprehensive representation of regulatory variants that associate with RNA expression (eQTL variants), or chromatin interaction and, consequently, improved our candidate gene mapping and pathway analysis. In addition, the consistency in the results between the GWAS of the binary and quantitative traits further confirms our findings. Most of the suggestive loci identified in the binary GWAS were also detected in the quantitative GWAS. Although our binary GWAS did not succeed in catching significant hits, additional categorization of samples based on anti-HEV antibody levels seemed to improve the phenotype precision, resulting in GWAS significant associations.

In conclusion, we reported several host genetic variants associated with anti-HEV antibody response. This study provides new evidence on the role of human genetic variation in HEV infection and emphasizes the importance of further replication and functional validation.

### Limitations of the study

There are several main limitations in this study. First, our classification of cases and controls was based on anti-HEV IgG positivity only. This could have led to the misclassification of individuals previously exposed to HEV, whose antibody level has waned to undetectable levels. Until now, the longevity of immunological response following HEV infection remains unclear. Multiple small-scale early and recent reports showed that anti-HEV antibodies could persist from 5 to 30 years post-infection in an age-dependent manner.^53^ Importantly, 80% of individuals were still positive for anti-HEV antibodies at least 10 years post-infection.^54^ Nonetheless, this does not eliminate the possibility of having false negative cases in our control group. Our results, therefore, provide insights on variants related to the antibody response and perhaps correlate with durability but cannot be translated into susceptibility due to the lack of solid evidence of exposure. Moreover, although we performed two GWASs, our results require replication in an entirely different cohort. It was also unfeasible to perform a meta-analysis due to the unavailability of other GWASs on HEV infection.

## STAR★Methods

### Key resources table

**Table:** 

REAGENT or RESOURCE	SOURCE	IDENTIFIER
**Biological samples**		

Serum samples	Qatar Biobank (QBB)	N/A

**Critical commercial assays**		

HEV ELISA-IgG	MP Biomedicals	SKU: 0721150096T

**Software and algorithms**		

PLINK-1.9.	Purcell et al.^55^	http://pngu.mgh.harvard.edu/purcell/plink/
SAIGE (Scalable and Accurate Implementation of GEneralized mixed model)	Zhou et al.^56^	https://github.com/weizhouUMICH/SAIGE/
Functional Mapping and Annotation of genetic associations (FUMA)	Watanabe et al.^57^	https://fuma.ctglab.nl/
Web-based Gene SeT AnaLysis Toolkit	Wang et al.^19^	http://www.webgestalt.org
R (version 4.2.1)	The R Foundation	www.R-project.org

**Other**		

Whole genome sequences	Qatar Biobank (QBB)	N/A

### Resource availability

#### Lead contact

Further information and requests for resources and reagents should be directed to and will be fulfilled by the Lead Contact, Maria K. Smatti (msmatti@qu.edu.qa).

#### Materials availability

The materials used in this paper (biological samples and whole genome sequences) are owned by Qatar Biobank (https://www.qatarbiobank.org.qa).

### Experimental model and study participant details

#### Sample selection criteria

The present study included a cohort from Qatar Biobank (QBB) participants. Whole-genome sequences of 6,218 Qatari nationals who had previously participated in phase 1 of the Qatar Genome Project (QGP) were obtained. This cohort represented the first data release from QBB. However, the corresponding sera samples were available for only 6000 individuals. A detailed demographic characterization of this cohort has been previously documented.^58^ QBB recruits adults (age ≥ 18 years) following specific inclusion/exclusion criteria to comprehensively include the permanent heterogeneous population in Qatar. Only Qatari nationals or long-term residents (≥ 15 years living in Qatar) are eligible to participate. All study participants were of Qatari origin. Male participants represented 43.2% (n=2593) of the study population compared to 56.8% (n=3407) females. The mean age of enrolled subjects was 40.5 years (SD±12). All QBB participants signed informed consent before their participation.

#### Ethical approval

The study was approved by Qatar University Institutional Review Board (IRB, QU-IRB 1287-EA/20) and Qatar Biobank IRB (E-2020-QBB-RES-ACC-0184-0110).

### Method details

#### Genomic data and quality control

Sequencing read data were generated by Illumina HiSeq X Ten sequencers and converted from the native BCL format to paired-end FASTQ format using bcl2fastq. The quality of the raw data was assessed using fastqc.^59^ Data passing quality control was then aligned to the reference genome sequence (build GRCh37 (hs37d53) using the bwa-kit6 aligner [v0.7.12].^60^ Variant calling was performed using GATK haplotype caller [v3.3].^61^^,^^62^ The genetic variant data was then converted to PLINK file format using PLINK-1.9.^55^ Standardized quality-assurance and quality control (QA/QC) methods were followed to generate high quality and confidence on both SNPs and sample levels, as previously described.^58^ Briefly, SNPs with genotype call rate <90%, Hardy-Weinberg p-value <1x10^-6^ were removed. Additionally, subjects with excess heterozygosity, duplicates, call rate <95%, and gender ambiguity were removed. We calculated the Pairwise identity by-state (IBS) matrix based on a pruned set of independent autosomal SNPs (N = 62,475) using a window size of 200 SNPs and LD threshold of r2 = 0.05 to identify and remove duplicated samples. Moreover, multidimensional scaling (MDS) was performed to identify and remove population ancestry outliers. Samples with ±4 SD from the mean of the two MDS components were considered population outliers and removed from the analysis.^52^^,^^58^ The final set of sequences used for the GWAS comprised 8,408,727 SNPs. The pass QC number of samples was 6045.

#### Binary and quantitative phenotypes classification

Sera samples (n=6000) were tested for HEV IgG antibodies using a commercial ELISA assay (HEV ELISA-IgG, MP Biomedicals) according to the manufacturer's protocol. For the binary trait GWAS, samples were grouped into cases or controls based on the anti-HEV IgG positivity. For the quantitative GWAS, samples were semi-quantitatively categorized into five groups based on their ELISA absorbance value, which correlates with the anti-HEV antibody level.^63^ We used the positive control value as a reference because it is plate/experiment-specific. Samples were classified as follows: Group1= samples with absorbance higher than 1.5∗(positive control); Group2= samples with absorbance higher than the positive control but less than 1.5∗(positive control); Group3= samples with absorbance higher than the positive control and higher than 0.5∗(positive control); Group5= positive samples with absorbance less than or equal to 0.5∗(positive control); and Group 6= negative samples. Two samples were excluded due to indeterminate ELISA results obtained from two separate experiments. As per the phenotypic data availability and genomic QC filtration, the final total number of subjects included in the GWAS was 5829 individuals.

### Quantification and statistical analysis

#### Genome-wide association analysis

Genome-wide association testing was performed using the SAIGE (Scalable and Accurate Implementation of GEneralized mixed model) implemented in the R package.^56^ This tool is based on a generalized mixed model association test that uses the saddlepoint approximation to calibrate the distribution of score test statistics for binary and quantitative traits. Importantly, this method provides better association accuracy because it accounts for case-control imbalance and sample relatedness.^56^ For all tested traits, we included age, sex, and the first four principal components (PCs) as covariates in the model. Principal component analysis was performed using PLINK. The genome-wide significance threshold was set as *p*<5×10^−8 28^. Quantile-Quantile plots, Manhattan plots, and regional association plots were all generated using Functional Mapping and Annotation of genetic associations (FUMA).^57^

#### Functional annotation

Functional annotation was performed using the FUMA platform (https://fuma.ctglab.nl/), an integrative web-based tool that processes GWAS summary statistics for functional annotation and gene prioritization. First, a pre-calculated LD structure based on the 1000G reference population was used to define a genomic risk locus. Independent significant variants (p<5x10^-8^) which are independent of each other at r^2^ < 0.6 were also defined. Additionally, SNPs that are independent at r^2^ < 0.1 were considered independent lead SNPs. Moreover, all SNPs in LD with an independent SNP (even if not in the GWAS results) are added to the annotation.^57^ Variant annotation with biological functionality and gene mapping was then carried out according to three main strategies: physical position, expression quantitative trait loci (eQTL), and chromatin interaction. FUMA includes 18 biological data repositories for gene-set analysis by MAGMA (Multi-marker Analysis of GenoMic Annotation). MAGMA applies multiple linear regression models to examine whether genes in different gene sets are strongly associated with a trait. This was followed by gene-property analysis to investigate further the relationship between tissue-specific gene expression profiles and gene associations.^64^ Gene analysis was performed using MAGMA (v1.6) with default settings. Significant p-value threshold set at 0.05 / (number of tested genes).

#### Gene enrichment analysis

Gene enrichment analysis was performed utilizing WebGestalt (Web-based Gene SeT AnaLysis Toolkit, http://www.webgestalt.org). This toolkit integrates functional categories from different databases and computational analyses to provide a comprehensive yet easy way to evaluate gene sets for biological context interpretation.^19^ We used the over-representation analysis (ORA) method for Gene ontology (GO), pathways (Reactome), and disease (GLAD4U) databases. For all analyses, the number of genes in each pathway was set from 5 to 2000, and Benjamini-Hochberg FDR was used for multiple test adjustment. Furthermore, InnateDB database (https://www.innatedb.com) was used to determine which genes are involved in immune-relevant pathways. Briefly, the combined set of mapped genes from the quantitative and qualitative GWASs (n=1866) was filtered to select protein-coding genes only (n=537). This list was then annotated using InnateDB annotation tool to explore which genes are associated with immune responses. Subsequently, the resulting list of genes that have an “immune response” function (n=44) was subjected to functional enrichment analysis.

## Data Availability

• Whole-genome sequence data are not publicly available in repositories. To obtain access, an application must be submitted to the Qatar Biobank. • This paper does not report original code. • Results of the study (raw summary statistics) and any additional information required to reanalyze the data reported in this paper are available from the lead contact upon request.

## Supplemental information

Document S1. Figures S1 and S2 and Tables S1, S2, S4, and S6–S9

Table S3. List of genes mapped to gnomic loci associated with HEV seropositivity binary GWAS related to Figures 1 and 2 Footnote: ENSG: Ensembl gene IDs; posMapSNPs (posMap): The number of SNPs mapped to gene based on positional mapping; eqtlMapSNPs: The number of SNPs mapped to the gene based on eQTLs mappingg; ciMap: "Yes" if the gene is mapped by chromatin interaction mapping.

Table S5. List of genes mapped eQTLs from the HEV seropositivity quantitative GWAS related to Figures 1 and 2 Footnote: ENSG: Ensembl gene IDs; eqtlMapSNPs: The number of SNPs mapped to the gene based on eQTLs mappingg; eqtlMapminP (eqtlMap): The minimum eQTL P-value of mapped SNPs. IndSigSNPs: rsID of the all independent significant SNPs of mapped SNPsList of figure titles and legends.
