# Supplementary material for: Genome-wide association study identifies several loci for HEV seropositivity
Source: iScience. 2023 Aug 10;26(9):107586. doi: 10.1016/j.isci.2023.107586 (PMC10470371; doi:10.1016/j.isci.2023.107586)
Supplement: Document S1. Figures S1 and S2 and Tables S1, S2, S4, and S6–S9 [file mmc1.pdf]

iScience, Volume *26*

Supplemental information

Genome-wide association study identifies several loci for HEV seropositivity

Maria K. Smatti, Yasser A. Al-Sarraj, Omar Albagha, and Hadi M. Yassine

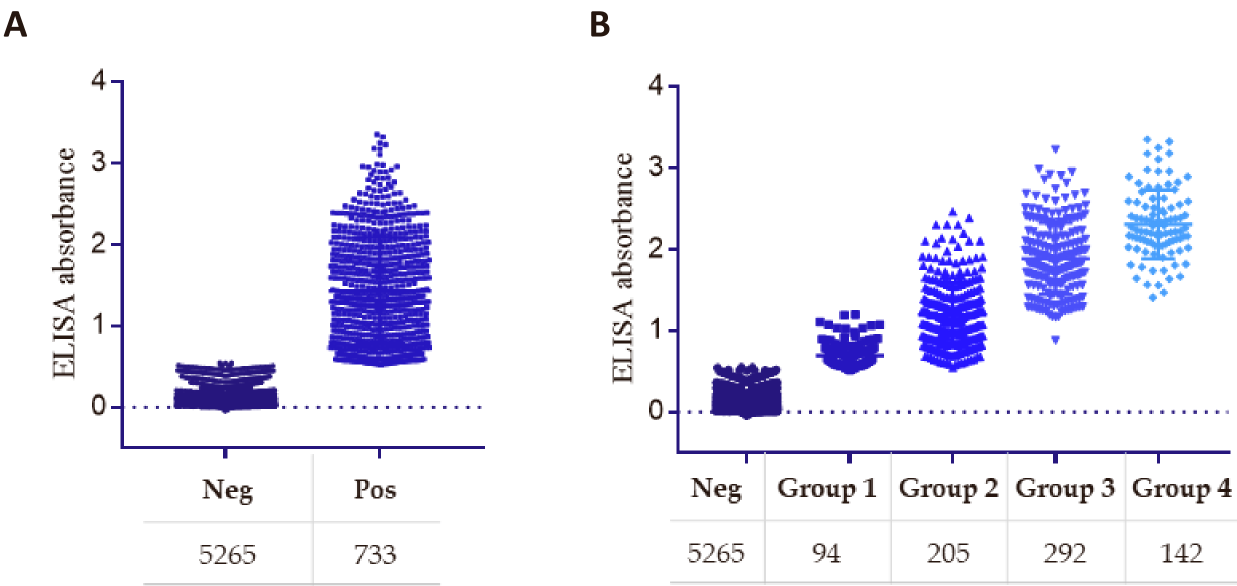

Neg	Pos
5265	733

Neg	Group 1	Group 2	Group 3	Group 4
5265	94	205	292	142

Supplementary Figure 1. Representation of anti-HEV IgG ELISA results, related to Table 1. A. Binary HEV ELISA results. Samples (n=6000) were classified positive or negative for anti-HEV based on the ELISA absorbance results. **B.** Categorical classification of positive samples to ordinal groups**.** Positive samples (n=733) were further classified to four groups based on the ELISA absorbance. The scattered plots show the number of samples in each group, which corresponds to the numbers in the table.

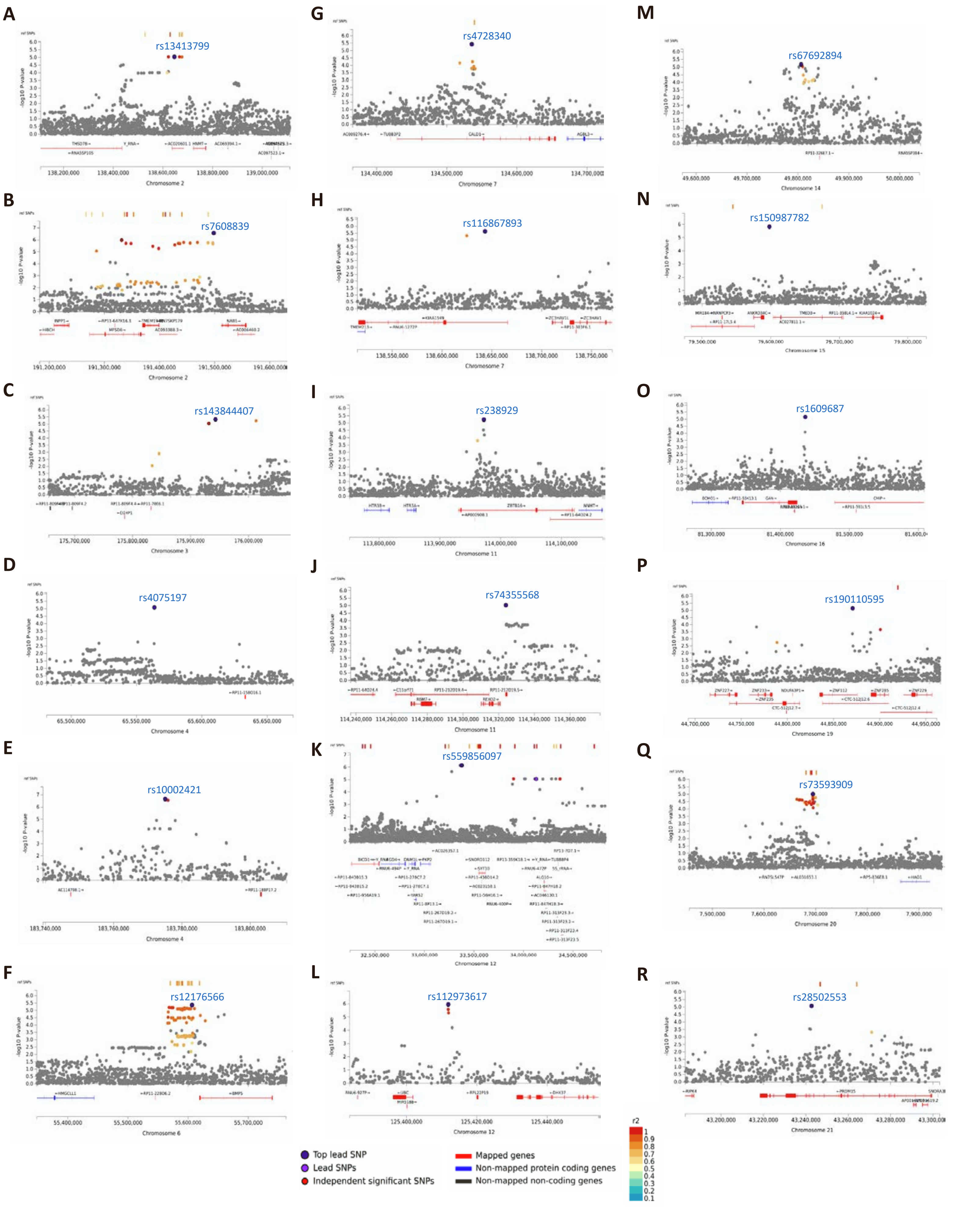

Supplementary Figure 2. Regional association plots for 18 genomic loci suggestively associated with HEV seropositivity, related to Figures 1 and Figure 2. The top lead SNP at each suggestive genomic locus is labeled with the rs identifier. FUMA platform (https://fuma.ctglab.nl/) was used for generating the regional plots.

Table S1. Unique candidate SNPs in LD with the lead SNP (rs559856097) associated with anti-HEV level quantitative GWAS, related to Figure 3

No	rsID	Chr	Pos	A1	A2 (effect)	GWAS p-value	Beta	r2	Ind. Sig. SNP	Nearest Gene	Distance	Function	CADD
1	rs559856097	12	33357549	G	A	2.30E-10	-0.2766	1	rs559856097	SNORD112	157968	intergenic	12.6
2	rs372352546	12	33873386	A	G	6.54E-08	-0.2771	1	rs559856097	RP13-359K18.1	2233	intergenic	0.019
3	rs371520150	12	34336088	G	A	6.85E-08	-0.3127	1	rs559856097	RP11-313F23.4	7883	intergenic	1.859
4	rs536434393	12	32363128	G	T	NA	NA	1	rs559856097	BICD1	0	intronic	2.331
5	rs569968872	12	32388689	C	G	NA	NA	1	rs559856097	BICD1	0	intronic	0.162
6	rs548415407	12	32443544	T	C	NA	NA	1	rs559856097	BICD1	0	intronic	12.05
7	rs368940180	12	33186766	C	T	NA	NA	1	rs559856097	AC026357.1	7892	intergenic	0.388
8	rs187953891	12	33221372	T	A	NA	NA	0.79	rs559856097	AC026357.1	26616	intergenic	2.21
9	rs375155014	12	33519560	C	G	NA	NA	1	rs559856097	SNORD112	3969	intergenic	4.519
10	rs375728624	12	33521475	C	G	NA	NA	1	rs559856097	SYT10	5697	intergenic	0.189
11	rs372866744	12	33525345	C	A	NA	NA	1	rs559856097	SYT10	1827	intergenic	0.92
12	rs539747309	12	33538189	C	T	NA	NA	1	rs559856097	SYT10	0	exonic	27.5
13	rs577535277	12	33880620	T	C	NA	NA	1	rs559856097	RP13-359K18.1	9467	intergenic	1.408
14	rs375259951	12	34073866	C	T	NA	NA	1	rs559856097	RNU6-472P	2442	intergenic	1.195
15	rs376018573	12	34098499	C	T	NA	NA	1	rs559856097	RNU6-472P	22083	intergenic	0.973
16	rs367759570	12	34336079	T	A	NA	NA	1	rs559856097	RP11-313F23.4	7892	intergenic	1.691

Chr: Chromosome number; Pos: Genomic position in human genome hg19; A1: Allele1; A2, Allele2 which is the effect allele; Ind.Sig.SNP: Independent significant SNP; CADD: Combined Annotation Dependent Depletion score.

Table S2. Genomic loci suggestively associated with anti-HEV level quantitative GWAS (p<1x10-5) related to Figure 2

Genomic Locus	Unique ID	Top lead SNP	Chr	P-value	Start	End	SNPs	nGWAS SNPs	Ind Sig SNPs	Lead SNPs
1	1:110114192:A:G	rs114849051	1	3.98E-06	110106185	110167583	4	2	rs114849051	rs114849051
2	1:182115278:C:T	rs3845453	1	4.22E-06	182115278	182144112	2	2	rs3845453	rs3845453
3	1:196884743:C:G	rs6662876	1	5.76E-06	196832133	196982776	87	77	rs6662876	rs6662876
4	2:4284917:A:G	rs72780542	2	9.55E-06	4284917	4284917	1	1	rs72780542	rs72780542
5	2:69472419:C:T	rs72903177	2	2.16E-06	69467604	69472633	4	2	rs72903177	rs72903177
6	2:77838661:C:T	rs79156816	2	9.77E-06	77764377	77838661	3	2	rs79156816	rs79156816
7	2:111778564:C:T	rs10200225	2	2.76E-06	111778564	111778564	1	1	rs10200225	rs10200225
8	2:119987418:G:T	rs73948625	2	2.70E-06	119966103	120024441	23	9	rs73948625<br>rs76364110	rs73948625
9	2:152394005:A:G	rs17198052	2	9.42E-06	152293120	152473911	7	2	rs17198052	rs17198052
10	2:191331304:C:G	rs60915953	2	2.82E-06	191265520	191498404	62	48	rs60915953<br>rs7608839	rs60915953
11	3:1079112:C:T	rs76191034	3	4.03E-06	1056386	1090860	10	8	rs76191034	rs76191034
12	3:32362718:A:G	rs56325818	3	7.94E-06	32362718	32369178	2	2	rs56325818	rs56325818
13	3:75293819:G:T	rs9310320	3	8.28E-06	74879971	75293819	7	7	rs9310320	rs9310320
14	3:175943035:A:T	rs143844407	3	1.78E-06	175833738	176013086	5	5	rs143844407<br>rs190707108	rs143844407
15	4:163828322:C:T	rs146895876	4	1.42E-06	163828322	163828322	1	1	rs146895876	rs146895876
16	4:183775102:A:G	rs10002421	4	6.08E-08	183765274	183784195	19	16	rs10002421<br>rs35935372<br>rs58931823	rs10002421
17	5:21170091:C:G	rs545105644	5	7.75E-06	20985606	21170091	4	4	rs545105644	rs545105644
18	5:77533703:A:G	rs75723200	5	5.71E-06	77468763	77533703	2	1	rs75723200	rs75723200
19	6:39636727:C:T	rs139341049	6	3.84E-06	39636727	39706983	2	2	rs139341049	rs139341049
20	6:55606378:C:T	rs12176566	6	1.44E-07	55565836	55659916	127	107	rs12176566<br>rs7750662	rs12176566
21	6:153216826:A:C	rs561981492	6	3.53E-06	153216826	153216826	1	1	rs561981492	rs561981492
22	6:168050422:C:T	rs139770629	6	2.19E-06	168050422	168050422	1	1	rs139770629	rs139770629
23	7:11839054:C:T	rs6972240	7	6.18E-06	11839054	11839054	1	1	rs6972240	rs6972240

24	7:138643158:A:T	rs116867893	7	1.77E-06	138624908	138643158	2	2	rs116867893	rs116867893
25	9:109822617:A:G	rs189645505	9	2.03E-06	108851004	110730995	17	1	rs189645505	rs189645505
26	9:120353505:A:G	rs7857205	9	5.77E-06	120346282	120356104	10	8	rs7857205	rs7857205
27	9:130450660:C:T	rs36089752	9	2.93E-06	130329786	130450660	3	3	rs36089752	rs36089752
28	10:881575:C:T	rs140582246	10	3.31E-06	881575	915555	2	2	rs140582246	rs140582246
29	10:6166400:C:T	rs7897142	10	7.13E-06	6166400	6166400	1	1	rs7897142	rs7897142
30	10:48550974:C:G	rs9421838	10	9.89E-06	48533604	48550974	5	1	rs9421838	rs9421838
31	10:76888065:A:G	rs377009718	10	2.36E-06	76845549	76888065	5	3	rs377009718	rs377009718
32	11:301809:C:T	rs147983088	11	4.90E-06	301809	301813	2	2	rs147983088	rs147983088
33	11:113972439:A:G	rs238929	11	5.92E-06	113962011	113972439	2	2	rs238929	rs238929
34	11:114324060:A:T	rs74355568	11	1.72E-06	114324060	114324060	1	1	rs74355568	rs74355568
35	12:28098719:C:T	rs80322123	12	1.59E-06	28086952	28112410	4	4	rs80322123	rs80322123
36	12:33357549:A:G	rs559856097	12	2.30E-10	32363128	34671065	24	5	rs559856097 rs191590523 rs77221465	rs559856097 rs77221465
37	12:38476689:C:T	rs531329952	12	8.23E-06	38162563	39417865	9	1	rs531329952	rs531329952
38	12:125411886:G:T	rs112973617	12	8.11E-07	125411872	125411975	3	3	rs112973617	rs112973617
39	14:21050601:A:G	rs113022222	14	9.25E-07	21050601	21050601	1	1	rs113022222	rs113022222
40	14:22765036:C:T	rs112971093	14	9.75E-06	22765036	22767841	2	2	rs112971093	rs112971093
41	14:69474330:C:T	rs150610998	14	1.67E-06	69474330	69474330	1	1	rs150610998	rs150610998
42	14:73187209:C:T	rs113520307	14	7.43E-06	73187209	73195598	4	3	rs113520307	rs113520307
43	15:79598147:A:T	rs150987782	15	2.05E-06	79543932	79674378	3	1	rs150987782	rs150987782
44	16:20245520:C:T	rs192237801	16	9.02E-06	20245520	20245520	1	1	rs192237801	rs192237801
45	16:82636475:A:G	rs11150480	16	5.46E-06	82636321	82636475	3	3	rs11859234 rs11150480	rs11859234 rs11150480
46	16:86473311:C:T	rs79169955	16	6.20E-06	86471147	86473311	2	2	rs79169955	rs79169955
47	17:57133429:G:T	rs77938575	17	7.93E-06	56625611	57133429	13	11	rs77938575	rs77938575
48	18:14192006:A:G	rs11875695	18	1.23E-06	14190168	14244421	30	22	rs11875695	rs11875695
49	18:20661514:G:T	rs139036753	18	2.10E-06	20661514	20672628	2	1	rs139036753	rs139036753
50	18:48588855:C:T	rs567553229	18	5.79E-06	47640112	49487533	8	1	rs567553229	rs567553229
51	18:51046131:G:GAATA	rs10531273	18	4.97E-06	50911436	51059163	47	33	rs10531273	rs10531273

52	19:32511363:C:G	rs1014367	19	2.86E-06	32473491	32515088	74	66	rs1014367	rs1014367
53	19:50224892:A:G	rs79890159	19	9.32E-06	50224892	50224892	1	1	rs79890159	rs79890159
54	20:5057691:C:T	rs150040846	20	2.04E-06	5057691	5057691	1	1	rs150040846	rs150040846
55	20:56018119:C:T	rs139019267	20	3.99E-06	56018119	56018119	1	1	rs139019267	rs139019267
56	21:42593863:A:G	rs141087220	21	9.92E-06	42593863	42593863	1	1	rs141087220	rs141087220

uniqID: Unique ID of SNPs consisting of chr:position:allele1:allele2 where alleles are alphabetically ordered (in human genome hg19); Chr: Chromosome number; start: Start position of the locus; end: End position of the locus; nGWAS SNPs: The number of unique GWAS-tagged candidate SNPs in the genomic locus

Table S4. List of genes mapped to the lead SNP (rs559856097) associated with anti-HEV level quantitative GWAS, related to Figure 3

No	ENSG	Symbol	Type	posMapSNPs	eqtlMapSNPs	ciMap
1	ENSG00000252390	RNU5F-4P	snRNA	0	0	Yes
2	ENSG00000207477	RNU6-318P	snRNA	0	0	Yes
3	ENSG00000212475	RNU6-400P	snRNA	0	0	Yes
4	ENSG00000207026	RNU6-472P	snRNA	2	0	Yes
5	ENSG00000200388	RNU6-618P	snRNA	0	0	Yes
6	ENSG00000251863	SNORD112	snoRNA	4	0	Yes
7	ENSG00000238661	snoU13	snoRNA	0	0	Yes
8	ENSG00000239033	snoU13	snoRNA	0	0	Yes
9	ENSG00000272435	5S_rRNA	rRNA	2	0	Yes
10	ENSG00000233381	AK4P3	pseudogene	0	0	Yes
11	ENSG00000257316	RP11-267D19.2	pseudogene	2	0	Yes
12	ENSG00000257511	RP11-278C7.1	pseudogene	0	0	Yes
13	ENSG00000256986	RP11-313F23.2	pseudogene	2	0	Yes
14	ENSG00000258794	RP11-313F23.5	pseudogene	2	0	No
15	ENSG00000244436	RP11-428G5.1	pseudogene	0	0	Yes
16	ENSG00000242314	RP11-428G5.2	pseudogene	0	0	Yes
17	ENSG00000256465	RP11-428G5.6	pseudogene	0	0	Yes
18	ENSG00000256987	RP11-428G5.7	pseudogene	0	0	Yes
19	ENSG00000256843	RP11-467L13.4	pseudogene	0	0	Yes
20	ENSG00000223722	RP11-467L13.5	pseudogene	0	0	Yes
21	ENSG00000257094	RP11-50I19.1	pseudogene	0	0	Yes
22	ENSG00000256070	RP11-56H16.1	pseudogene	4	0	Yes
23	ENSG00000243517	RP11-627K11.1	pseudogene	0	0	Yes
24	ENSG00000256176	RP11-627K11.3	pseudogene	0	0	Yes
25	ENSG00000270395	RP11-627K11.4	pseudogene	0	0	Yes
26	ENSG00000270766	RP11-627K11.5	pseudogene	0	0	Yes
27	ENSG00000203437	RP11-820K3.3	pseudogene	0	0	Yes
28	ENSG00000256159	RP11-820K3.4	pseudogene	0	0	Yes
29	ENSG00000257852	RP11-843B15.1	pseudogene	0	0	Yes
30	ENSG00000271357	RP11-843B15.3	pseudogene	0	0	Yes
31	ENSG00000244743	RP11-8P13.1	pseudogene	0	0	Yes
32	ENSG00000258134	RP11-956A19.1	pseudogene	3	0	Yes
33	ENSG00000270807	RP13-359K18.1	pseudogene	2	0	Yes

34	ENSG00000256614	RP13-7D7.1	pseudogene	2	0	No
35	ENSG00000231788	RPL31P50	pseudogene	0	0	Yes
36	ENSG00000250788	TUBB8P4	pseudogene	2	0	Yes
37	ENSG00000177340	AC024940.1	protein_coding	0	0	Yes
38	ENSG00000139133	ALG10	protein_coding	1	0	Yes
39	ENSG00000151743	AMN1	protein_coding	0	0	Yes
40	ENSG00000151746	BICD1	protein_coding	3	0	Yes
41	ENSG00000170456	DENND5B	protein_coding	0	0	Yes
42	ENSG00000087470	DNM1L	protein_coding	0	0	Yes
43	ENSG00000139146	FAM60A	protein_coding	0	0	Yes
44	ENSG00000174718	KIAA1551	protein_coding	0	0	Yes
45	ENSG00000139160	METTL20	protein_coding	0	0	Yes
46	ENSG00000187950	OVCH1	protein_coding	0	0	Yes
47	ENSG00000057294	PKP2	protein_coding	0	0	Yes
48	ENSG00000110975	SYT10	protein_coding	4	0	Yes
49	ENSG00000110900	TSPAN11	protein_coding	0	0	Yes
50	ENSG00000139131	YARS2	protein_coding	0	0	Yes
51	ENSG00000201228	Y_RNA	misc_RNA	0	0	Yes
52	ENSG00000201624	Y_RNA	misc_RNA	2	0	Yes
53	ENSG00000252584	AC023050.1	miRNA	0	0	Yes
54	ENSG00000266482	AC023157.1	miRNA	0	0	Yes
55	ENSG00000265029	AC023158.1	miRNA	4	0	Yes
56	ENSG00000238911	AC026357.1	miRNA	2	0	Yes
57	ENSG00000264446	AC046130.1	miRNA	1	0	Yes
58	ENSG00000257435	RP11-267D19.1	lincRNA	2	0	Yes
59	ENSG00000255628	RP11-313F23.3	lincRNA	2	0	Yes
60	ENSG00000255652	RP11-313F23.4	lincRNA	2	0	No
61	ENSG00000255760	RP11-428G5.5	lincRNA	0	0	Yes
62	ENSG00000259937	RP11-438D14.2	lincRNA	4	0	Yes
63	ENSG00000256232	RP11-771K4.1	lincRNA	0	0	Yes
64	ENSG00000256538	RP11-847H18.3	lincRNA	1	0	Yes
65	ENSG00000255867	DENND5B-AS1	antisense	0	0	Yes
66	ENSG00000257456	RP11-310I24.1	antisense	0	0	Yes
67	ENSG00000257530	RP11-843B15.2	antisense	0	0	Yes
68	ENSG00000245482	RP11-847H18.2	antisense	1	0	Yes

ENSG: Ensembl gene IDs; posMapSNPs (posMap): The number of SNPs mapped to gene based on positional mapping; eqtlMapSNPs: The number of SNPs mapped to the gene based on eQTLs mappingg; ciMap: "Yes" if the gene is mapped by chromatin interaction mapping.

Table S6. Top MAGMA pathways identified in the HEV seropositivity binary GWAS, related to Figure 4

Gene Set	No of genes	Beta	Beta STD	SE	P-value	P_{bon}
Curated_gene_sets:nemeth_inflammatory_response_lps_up	82	0.377	0.024551	0.086839	7.13E-06	0.110346726
Curated_gene_sets:burton_adipogenesis_peak_at_24hr	46	0.50715	0.02476	0.12536	2.62E-05	0.406010709
Curated_gene_sets:pid_smad2_3pathway	16	0.73181	0.021088	0.19204	6.96E-05	1
GO_mf:go_molecular_carrier_activity	40	0.51809	0.02359	0.13668	7.54E-05	1
Curated_gene_sets:goldrath_homeostatic_proliferation	137	0.26968	0.022668	0.073041	0.00011157	1
GO_bp:go_regulation_of_membrane_repolarization_during_ventricular_cardiac_muscle_cell_action_potential	4	1.5534	0.022388	0.4213	0.00011379	1
GO_bp:go_regulation_of_membrane_repolarization_during_cardiac_muscle_cell_action_potential	4	1.5534	0.022388	0.4213	0.00011379	1
Curated_gene_sets:goldrath_antigen_response	333	0.17579	0.022918	0.047685	0.00011409	1
Curated_gene_sets:osawa_tnf_targets	10	0.94346	0.021496	0.26259	0.00016409	1
Curated_gene_sets:reactome_pi3k_events_in_erbb2_signaling	16	0.79725	0.022973	0.22246	0.00016991	1

Beta: the regression coefficient of the variable; Beta_STD: the semi-standardized regression coefficient, corresponding to the predicted change in Z-value given a change of one standard deviation in the predictor gene set / gene covariate; SE: the standard error of the regression coefficient; Pbon: Bonferroni corrected p-value.

Table S7. Top MAGMA genes identified from the HEV seropositivity binary GWAS related to Figure 4

No	Gene	Chr	Start	Stop	No SNPs	Nparm	Zstat	P-value	Symbol
1	ENSG00000107036	9	5604025	5801557	459	50	3.5346	2.04E-04	KIAA1432
2	ENSG00000134460	10	6027652	6129288	409	81	3.5288	2.09E-04	IL2RA
3	ENSG00000134453	10	6105950	6184420	369	71	3.4631	2.67E-04	RBM17
4	ENSG00000099617	19	1261153	1326430	145	48	3.4634	2.67E-04	EFNA2
5	ENSG00000131899	17	18103901	18173189	196	29	3.3878	3.52E-04	LLGL1
6	ENSG00000170180	4	145005457	145086904	435	42	3.3505	4.03E-04	GYPA
7	ENSG00000185467	7	98746197	98830129	290	24	3.3465	4.09E-04	KPNA7
8	ENSG00000134365	1	196794371	196913102	229	22	3.338	4.22E-04	CFHR4
9	ENSG00000146021	5	136928189	137096779	335	50	3.3318	4.31E-04	KLHL3
10	ENSG00000080910	1	196763898	196953356	325	29	3.3304	4.34E-04	CFHR2
11	ENSG00000155542	5	56180087	56246359	191	20	3.3265	4.40E-04	SETD9
12	ENSG00000139926	14	51930818	52222445	920	150	3.2965	4.90E-04	FRMD6
13	ENSG00000120053	10	101131627	101215381	162	36	3.2903	5.00E-04	GOT1
14	ENSG00000180938	8	125960540	126016631	194	17	3.2681	5.41E-04	ZNF572
15	ENSG00000155545	5	56190429	56292502	326	38	3.25	5.77E-04	MIER3
16	ENSG00000154227	15	100915600	101110200	805	71	3.2347	6.09E-04	CERS3
17	ENSG00000213231	14	96127754	96183980	198	50	3.175	7.49E-04	TCL1B
18	ENSG00000198909	17	61674775	61798663	156	15	3.1743	7.51E-04	MAP3K3
19	ENSG00000147432	8	42527519	42617550	327	16	3.1643	7.77E-04	CHRNB3
20	ENSG00000198754	1	40210195	40262020	201	28	3.1444	8.32E-04	OXCT2

Chr: Chromosome number; Nparam: the number of relevant parameters used in the model; Zstat: the Z-value for the gene, based on its (permutation) p-value.

Table S8. Top MAGMA pathways identified in the anti-HEV level quantitative GWAS, related to Figure 4

Gene Set	No of genes	Beta	Beta STD	SE	P-value	P_{bon}
Curated_gene_sets:reactome_pi3k_events_in_erbb2_signaling	16	0.88055	0.025374	0.22304	0.000039608	0.613290272
GO_mf:go_nuclear_import_signal_receptor_activity	9	0.81559	0.01763	0.21132	0.000057037	0.883103871
Curated_gene_sets:reactome_irf3_mediated_induction_of_type_i_ifn	12	0.89663	0.022378	0.23409	0.000064277	0.995136514
GO_bp:go_modification_of_morphology_or_physiology_of_other_organism_involved_in_symbiotic_interaction	106	0.28754	0.021277	0.077173	0.000097674	1
GO_bp:go_modification_of_morphology_or_physiology_of_other_organism	153	0.25486	0.022629	0.068563	0.00010113	1
GO_bp:go_modification_by_symbiont_of_host_morphology_or_physiology	43	0.43294	0.020437	0.11648	0.00010122	1
GO_bp:go_postsynaptic_signal_transduction	9	0.87892	0.018998	0.23669	0.00010267	1
Curated_gene_sets:reactome_irf3_mediated_activation_of_type_1_ifn	5	1.1605	0.0187	0.31616	0.00012135	1
Curated_gene_sets:burton_adipogenesis_peak_at_24hr	46	0.45288	0.02211	0.12571	0.00015809	1
Curated_gene_sets:reactome_pi3k_events_in_erbb4_signaling	10	0.94087	0.021437	0.26157	0.00016146	1

Beta: the regression coefficient of the variable; Beta_STD: the semi-standardized regression coefficient, corresponding to the predicted change in Z-value given a change of one standard deviation in the predictor gene set / gene covariate; SE: the standard error of the regression coefficient; Pbon: Bonferroni corrected p-value.

Table S9. Top MAGMA genes identified from the anti-HEV level quantitative GWAS related to Figure 4

No	Gene	Chr	Start	Stop	No SNPs	Nparm	Zstat	P-value	Symbol
1	ENSG00000198909	17	61674775	61798663	156	15	3.6627	1.E-04	MAP3K3
2	ENSG00000136463	17	61653231	61710725	49	11	3.6119	2.E-04	TACO1
3	ENSG00000155542	5	56180087	56246359	191	20	3.539	2.E-04	SETD9
4	ENSG00000099617	19	1261153	1326430	145	48	3.5289	2.E-04	EFNA2
5	ENSG00000136490	17	61748262	61803532	82	11	3.5248	2.E-04	LIMD2
6	ENSG00000155545	5	56190429	56292502	326	38	3.5243	2.E-04	MIER3
7	ENSG00000146021	5	136928189	137096779	335	50	3.5144	2.E-04	KLHL3
8	ENSG00000147481	8	50797349	51731678	2554	128	3.5139	2.E-04	SNTG1
9	ENSG00000198754	1	40210195	40262020	201	28	3.499	2.E-04	OXCT2
10	ENSG00000134365	1	196794371	196913102	229	22	3.4922	2.E-04	CFHR4
11	ENSG00000080910	1	196763898	196953356	325	29	3.4757	3.E-04	CFHR2
12	ENSG00000076043	11	114285108	114346001	218	27	3.4587	3.E-04	REXO2
13	ENSG00000134453	10	6105950	6184420	369	71	3.4457	3.E-04	RBM17
14	ENSG00000074803	15	48458861	48621275	248	19	3.4328	3.E-04	SLC12A1
15	ENSG00000185467	7	98746197	98830129	290	24	3.4319	3.E-04	KPNA7
16	ENSG00000255663	11	114246404	114339654	307	41	3.4251	3.E-04	RP11-212D19.4
17	ENSG00000120053	10	101131627	101215381	162	36	3.4181	3.E-04	GOT1
18	ENSG00000176253	14	20477003	20527919	363	34	3.4082	3.E-04	OR4K13
19	ENSG00000124882	4	75205860	75279468	353	35	3.3941	3.E-04	EREG
20	ENSG00000176246	14	20503204	20554142	324	33	3.3882	4.E-04	OR4L1

Chr: Chromosome number; Nparam: the number of relevant parameters used in the model; Zstat: the Z-value for the gene, based on its (permutation) p-value.
